# Supplementary material for: Bisphenol A and Type 2 Diabetes Mellitus: A Review of Epidemiologic, Functional, and Early Life Factors
Source: Int J Environ Res Public Health. 2021 Jan 15;18(2):716. doi: 10.3390/ijerph18020716 (PMC7830729; doi:10.3390/ijerph18020716)
Supplement: Supplementary file 1 [file ijerph-18-00716-s001.pdf]

Figure S1

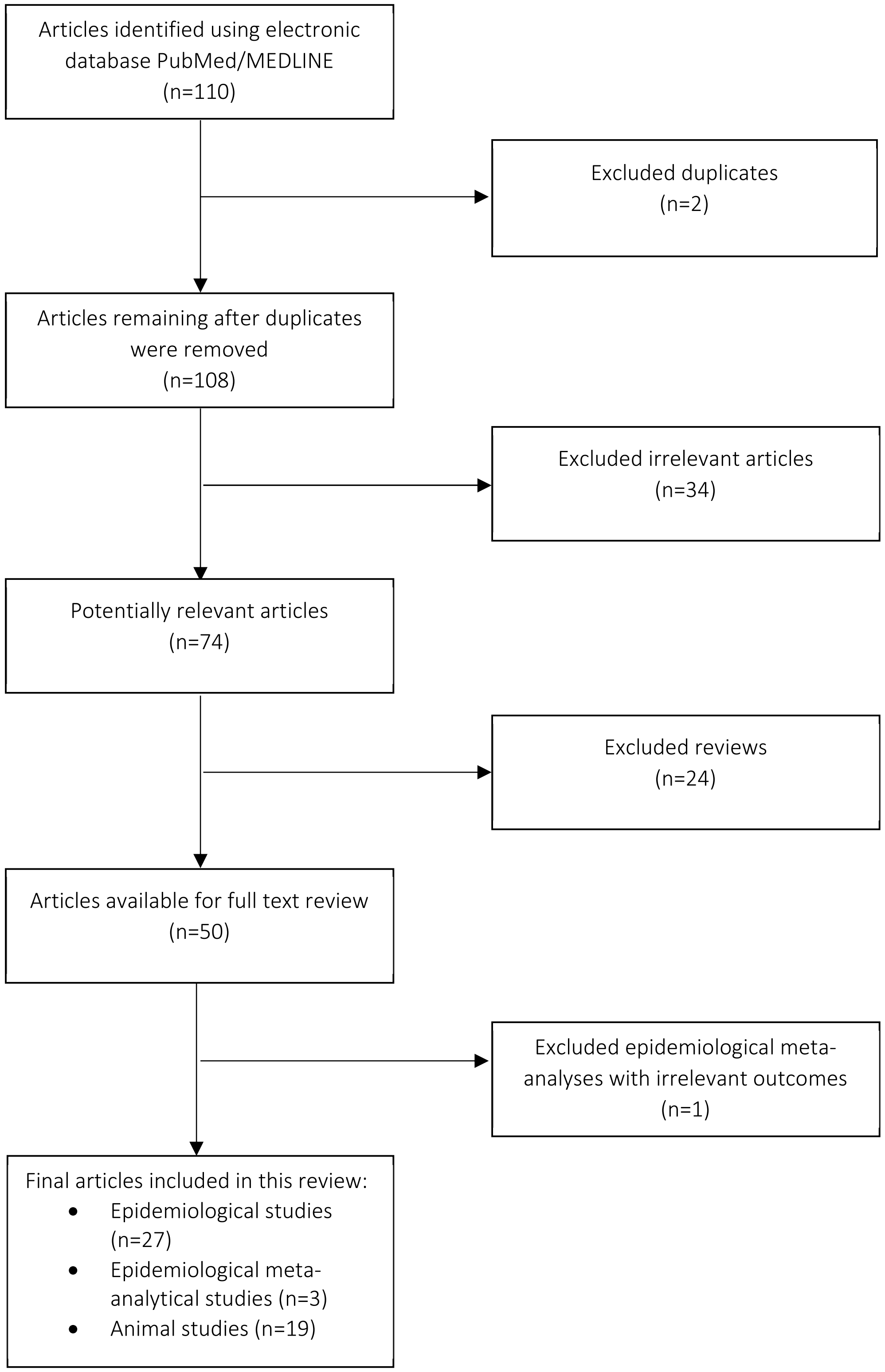

Figure S1. PRISMA flow diagram summarising the process of selecting articles to be used in this review.
